# Introduction of ORF3a-Q57H SARS-CoV-2 Variant Causing Fourth Epidemic Wave of COVID-19, Hong Kong, China

**DOI:** 10.3201/eid2705.210015

**Published:** 2021-05

**Authors:** Daniel K.W. Chu, Kenrie P.Y. Hui, Haogao Gu, Ronald L.W. Ko, Pavithra Krishnan, Daisy Y.M. Ng, Gigi Y.Z. Liu, Carrie K.C. Wan, Man-Chun Cheung, Ka-Chun Ng, John M. Nicholls, Dominic N.C. Tsang, Malik Peiris, Michael C.W. Chan, Leo L.M. Poon

**Affiliations:** The University of Hong Kong, Hong Kong, China (D.K.W. Chu, K.P.Y. Hui, H. Gu, R.L.W. Ko, P. Krishnan, D.Y.M. Ng, G.Y.Z. Liu, C.K.C. Wan, M.-C. Cheung, K.-C. Ng, J.M. Nicholls, M. Peiris, M.C.W. Chan, L.L.M. Poon);; Department of Health, Hong Kong (D.N.C. Tsang)

**Keywords:** respiratory infections, severe acute respiratory syndrome coronavirus 2, SARS-CoV-2, SARS, COVID-19, coronavirus disease, zoonoses, viruses, coronavirus, Hong Kong, China

## Abstract

We describe an introduction of clade GH severe acute respiratory syndrome coronavirus 2 causing a fourth wave of coronavirus disease in Hong Kong. The virus has an ORF3a-Q57H mutation, causing truncation of ORF3b. This virus evades induction of cytokine, chemokine, and interferon-stimulated gene expression in primary human respiratory cells.

Hong Kong, China, has had 4 waves of coronavirus disease (COVID-19) outbreaks since the emergence of severe acute respiratory syndrome coronavirus 2 (SARS-CoV-2) in December 2019. By February 1, 2021, Hong Kong had recorded 10,453 reverse transcription PCR (RT-PCR)–confirmed COVID-19 cases, and many of those occurred during the last 2 waves. The third wave occurred during late June to early September 2020 and was caused by a single introduction of GISAID (https://platform.gisaid.org) clade GR virus (*1*). The fourth wave began in early November 2020 and was caused by a newly introduced GISAID clade GH SARS-CoV-2 (*1*). We describe the origin of a clade GH virus causing the fourth epidemic wave in Hong Kong.

## The Study

**Figure 1:**
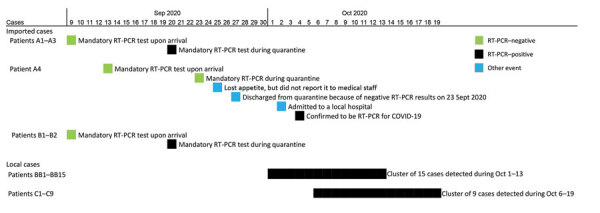
Timeline of COVID-19 cases during fourth epidemic, September 9–October 19, 2020, Hong Kong, China. Asymptomatic cases occurred in patients A1–A3, B1, B2, BB4, BB12, BB13, BB15, C3, C4, C7, and C9. Symptomatic cases occurred among patients A4, BB1–BB3, BB5–BB11, BB14, C1, C2, C5, C6, and C8. COVID-19, coronavirus disease; RT-PCR, reverse transcription PCR.

Before our investigation, epidemiologic investigations in early October 2020 revealed 2 local COVID-19 clusters associated with bar/building X or hotel C (Appendix Figure 1), both of which are located in the same district of Hong Kong. The bar/building X cluster had 15 RT-PCR–confirmed COVID-19 cases (patients BB1–BB15), and the hotel C cluster had 9 RT-PCR–confirmed cases (patients C1–C9) (Figure 1). 

To determine whether the 2 clusters were epidemiologically linked, we sequenced near full-length genomes from all available samples, including respiratory samples from patients BB1–BB13 and patients C1–C9, by using a previously described protocol (*2*,*3*). We found the viral genomes were highly similar (sequence identity >99.98%) (Appendix Figure 2). All sequences belonged to clade GH, which was not found in local COVID-19 cases during the third wave (*1*). Our results indicate that this newly introduced clade GH virus was circulating in the local community ≈1 month before the beginning of the fourth epidemic wave in Hong Kong.

We also noted 4 imported cases (patients A1–A4) in a nearby hotel (hotel A), which is ≈350 m walking distance from bar/building X and hotel C, during late September to early October 2020 (Figure 1; Appendix Table 1). Patients A1–A3 traveled from Nepal to Hong Kong on the same direct flight and had their mandatory quarantine in hotel A during September 9–20, 2020 (Appendix). Of note, 2 additional RT-PCR–confirmed cases, patients B1 and B2, traveled on the same flight as patients A1–A3 (Figure 1). B1 and B2 were unrelated to patients A1–A3 and had their mandatory quarantine in hotel B, which is in another district of Hong Kong. Our sequencing results indicate that the viral genomes of these 5 cases are identical or almost identical to those from the 2 local clusters (Appendix Figure 2). Although it is not known whether in-flight transmission occurred among patients A1–A3 and B1 and B2 (*2*), our results suggest that the fourth COVID-19 epidemic wave in Hong Kong was introduced from Nepal, and the deduced sequences are closely related to sequences from Nepal (Appendix Figure 2).

Patient A4, who was quarantined in hotel A during September 13–27, 2020, also traveled from Nepal to Hong Kong on a separate flight. The viral genome of case A4 is identical or closely related to sequences from patients A1–A3 and B1 and B2. Patient A4 had consecutive negative RT-PCR results upon arrival and on day 12 during quarantine (Figure 1). Patient A4 might have acquired SARS-CoV-2 in Nepal and had a long incubation period. Alternatively, A4 might have acquired the infection while quarantined in hotel A. We do not know how this virus was introduced into the local community. However, patient A4 finished the mandatory quarantine on September 27 and started to interact with the local community 7 days before testing positive for SARS-CoV-2. Patient A4 might have had opportunities to introduce the clade GH virus into the local community, but we cannot exclude the possibility that this virus was introduced in hotel A via an unnoticed transmission chain or chains.

Our full genome analysis revealed that the wave 4 virus has several nonsilent mutations associated with host adaptation (*4*–*6*; B. Zhou et al., unpub. data, https://doi.org/10.1101/2020.10.27.357558), including mutations in the RNA-dependent RNA polymerase (RdRp[L323P]), Spike(D614G), open reading frame 3a (ORF3a[Q57H]), ORF3b(E14*), and nucleocapsid (N[S194L]) proteins. The ORF3a(Q57H) mutation leads a major truncation of ORF3b protein, ORF3b(E14*) (*6*). Because the ORF3b protein is reported to be a potent interferon antagonist (*6*), we isolated the virus from patient A2 and conducted phenotypic characterizations using ex vivo human organ cultures and human airway organoids (*7*,*8*). We noted that this wave 4 virus contains a Spike(D614G) mutation that is associated with enhanced virus replication and transmission (B. Zhou et al., unpub. data). To differentiate the effect of Spike(D614G) and ORF3a(Q57H) mutations in our assays, we included viruses isolated from epidemic waves 1 and 3 as controls. The wave 1 virus we studied did not have these 2 mutations; the wave 3 virus had the Spike(D614G) but not the ORF3a(Q57H) mutation (Table). Our sequence data are available from GISAID (accession nos. EPI_ISL_760031–58).

**Table:** Amino acid differences between severe acute respiratory syndrome coronavirus 2 variants in 3 waves of coronavirus disease, Hong Kong, China*

Genome category	Amino acid position	Wave 1 virus	Wave 3 virus	Wave 4 virus
		VM20001061	Case 4533	Patient A2
ORF1A/1AB				
NSP2	141	M	**V**	M
NSP3	85	A	**V**	A
	238	V	V	**L**
	453	V	**I**	V
	1,179	A	A	**V**
RdRp	323	**P**	L	L
EndoRNAse	231	A	**V**	A
Spike	12	S	**F**	S
	25	**L**	P	P
	367	**F**	V	V
	614	**D**	G	G
	680	**Q**	R	R
	1,002	**E**	Q	Q
ORF3a	57	Q	Q	**H**
	227	T	T	**I**
ORF3b	14	E	E	STOP
ORF8	62	**L**	V	V
	84	S	L	L
Nucleocapsid	12	A	**G**	A
	194	S	S	L
	203	R	**K**	R
	204	G	**R**	G
ORF9b	9	H	**D**	H

*Bold text indicates position where isolated differs from the other isolates. A, alanine; D, aspartic acid; E, envelope; F, phenylalanine; G, glycine; H, histidine; I, isoleucine; L, leucine; M, membrane; NSP, nonstructural protein; ORF, open reading frame; P, proline; Q, glutamine; R, arginine; S, spike; STOP, stop codon; T, threonine; V, valine.

We first studied the virus replication kinetics by using human bronchus and lung ex vivo cultures (Appendix Figure 3). In bronchus tissues, the wave 4 virus had a replication rate comparable to the wave 1 virus, but it had a lower replication rate than the wave 1 virus in lung tissues at 48 h, 72 h, and 96 h and a lower area under the curve. By contrast, the wave 3 virus had a slightly higher replication rate than the wave 1 virus in human bronchus, but not in human lung ex vivo cultures (Appendix Figure 3, panel A). Immunohistochemical staining analyses confirmed these observations (Appendix Figure 3, panel B). We found the wave 3 virus, not the wave 4 virus, might have marginally better replication competence than the wave 1 virus.

**Figure 2:**
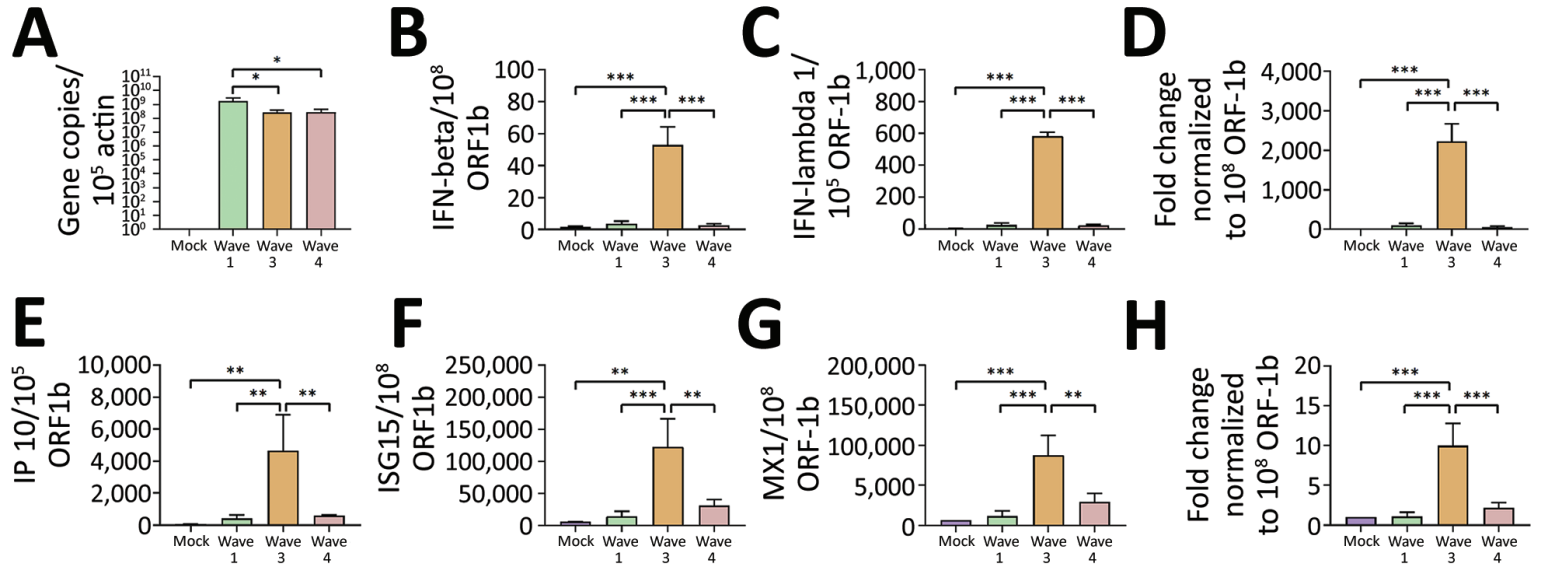
Innate immune responses in human airway organs experimentally infected with SARS-CoV-2 viruses from COVID-19 epidemic waves 1, 3, and 4, Hong Kong, China. A) ORF1b; B) IFN-β; C) IFN-λ 1; D) IFN-λ 2/3; E) IP-10; F) ISG15; G) MX1; H) MDA5. Messenger RNA expression of viral genes in human airway air-liquid interface organoids (n = 4; multiplicity of infection = 2) from the apical side at 48 h post infection. Mock samples were not infected. The gene expression of infected cells was first normalized with β-actin and further normalized with ORF1b gene. The gene expression of mock-infected cells was presented after normalization with β-actin. The differences were compared using 1-way ANOVA followed by a Tukey multiple-comparison test. Means and SD error bars are as shown. *p<0.05; **p<0.01; ***p<0.001. COVID-19, coronavirus disease; IFN, interferon; IP-10 interferon gamma-induced protein-10; ISG15, interferon stimulated gene 15; MDA5, melanoma differentiation-associated protein 5; MX1, interferon-induced GTP binding protein 1; ORF, open reading frame; SARS-CoV-2, severe acute respiratory syndrome coronavirus 2.

We previously demonstrated that the wave 1 virus is not a potent proinflammatory cytokine and chemokine inducer in infected human cells (*7*). To determine whether the ORF3a(Q57H) would affect this phenotype, we tested these viruses in human respiratory organoid cultures. We extracted RNA from infected organoids at 48 h post infection and tested the RNA samples in RT-PCR assays for a range of innate immune response genes. The viral RNA in organoids infected by the wave 3 or 4 virus was ≈1 log unit lower than the one infected by the wave 1 virus (p<0.05; Figure 2). The cytokine, chemokine, and interferon-stimulated gene mRNA levels induced by the wave 4 virus were low and were only similar to the wave 1 virus. In addition, gene expressions in cells infected by the wave 3 virus were much higher than those caused by the wave 1 virus. Interferon gamma-induced protein-10 measurement of these cultures corroborated our observations (Appendix Figure 4).

Despite the major ORF3b deletion, our results demonstrate that the wave 4 virus does not have an enhanced ability to replicate ex vivo and retains potent innate immune evasion capacity in our experimental models. We noted that the wave 3 virus replicates slightly better than isolates from wave 1 and 4, and it can induce higher innate immune responses. The wave 3 virus has several unique mutations not found in the other 2 viruses (Table). Many of these mutations are in the ORF1ab or N gene. Although not within the scope of this study, further characterization of mutations found in the wave 3 virus via reverse genetics (*9*) might help explain our observations.

### Conclusion

In summary, we found the virus causing the fourth COVID-19 epidemic wave in Hong Kong does not have enhanced replication kinetics and is not a potent cytokine or chemokine inducer. However, our work highlights the need for stringent COVID-19 control policy in quarantine settings.

Appendix Additional information on SARS-CoV-2 viruses from COVID-19 epidemic waves 1, 3, and 4, Hong Kong, China.
