# Supplementary material for: Introduction of ORF3a-Q57H SARS-CoV-2 Variant Causing Fourth Epidemic Wave of COVID-19, Hong Kong, China
Source: Emerg Infect Dis. 2021 May;27(5):1492–5. doi: 10.3201/eid2705.210015 (PMC8084491; doi:10.3201/eid2705.210015)
Supplement: Appendix — Additional information on SARS-CoV-2 viruses from COVID-19 epidemic waves 1, 3, and 4, Hong Kong, China. [file 21-0015-Techapp-s1.pdf]

Article DOI: https://doi.org/10.3201/eid2705.210015

Introduction of ORF3a-Q57H SARS-CoV-2 Variant Causing Fourth Epidemic Wave of COVID-19, Hong Kong, China

Appendix

Additional Methods

Quarantine Policy and Testing Strategy for Inbound Travelers

Inbound travelers arriving by air were tested by reverse transcription-PCR (RT-PCR) for severe acute respiratory syndrome coronavirus 2 (SARS-CoV-2) upon arrival at Hong Kong International Airport. Travelers were mandated to wait for results at the testing site in the airport. Positive coronavirus disease (COVID-19) cases were sent to a hospital for treatment, but negative cases stayed in a designated hotel for a 14-day compulsory quarantine. On day 12 of their quarantine period, quarantined travelers were instructed to provide samples for an additional RT-PCR test. All the RNA samples were tested in a World Health Organization (WHO) Reference Laboratory in the Public Health Laboratory Centre, Hong Kong. Current quarantine policy is available from https://www.coronavirus.gov.hk/eng/inbound-travel-faq.html.

Investigation of Local Cases

The investigation of local cases was initiated by the detection of an index case in each of the local clusters. Active contact tracing investigations were immediately conducted by the Hong Kong Government thereafter. Persons epidemiologically linked to these clusters (e.g., close contacts) were required to provide respiratory samples for COVID-19 RT-PCR tests. All epidemiologic data described in the manuscript were retrieved from public datasets (https://data.gov.hk/en-daata/dataset/hk-dh-chpsebcddr-novel-infectious-agent).

Sequencing

RNA samples for our study were sent to a WHO reference laboratory at the University of Hong Kong for full genome analyses (IRB no. UW 20–168). We deduced near full-length genomes from all available samples, patients BB1–BB13 and patients C1–C9, with sequence

length >29,700 nt and sequence coverage >100 by using an Illumina (https://www.illumina.com) sequencing protocol we described previously (*1*). In brief, virus genome was reverse transcribed with multiple gene-specific primers targeting different regions of the viral genome. The synthesized cDNA was then subjected to multiple overlapping 2-kb PCRs for full-genome amplification. PCR amplicons obtained from the same specimen were pooled and sequenced by using Nova sequencing platform (Illumina). Sequencing library was prepared by using Nextera XT (Illumina).

Phylogenetic Analysis

Generated sequencing reads were mapped to a reference virus genome by BWA (http://bio-bwa.sourceforge.net), and genome consensus was generated by Geneious version 11.1.4 (https://www.geneious.com). Sequences from each phylogenetic clade (G, GH, GR, L, O, S, and V) of SARS-CoV-2 were selected from GISAID for phylogenetic analysis (Appendix Table 2). Viral sequences were aligned by using BioEdit (https://www.bioedit.com) and phylogenetically analyzed by using MEGA-X (https://www.megasoftware.net). We constructed the phylogenetic tree by using the neighbor-joining method with 500 bootstraps.

Virus Isolates Used in Characterization

Virus isolated from the wave 1 (clade L), wave 3 (clade GR), and wave 4 (clade GH) of COVID-19 in Hong Kong were used for phenotypic characterization. The wave 1 virus was isolated from an imported case identified in a 39-year-old symptomatic man, as previously described (*2*). The wave 3 virus was isolated from a locally transmitted case in a 57-year-old symptomatic woman. The wave 4 virus was isolated from patient A2 in this study, a 43-year-old asymptomatic man. These patients recovered and were discharged from hospital.

Ex Vivo Cultures and Infection of Human Respiratory Tract

Infection procedures were performed as previously described (*2*). In brief, nontumor bronchus and lung tissues were obtained from patients undergoing elective surgery, as detailed previously (*3*,*4*). Fragments of human tissues were infected with each virus at 5×10^5 $TCID_{50}$/mL for 1 h at 37°C. The explants were washed 3 times with phosphate buffered saline (PBS) and placed in culture medium (F-12K nutrient mixture with L-glutamine and antibiotics) and incubated at 37°C with 5% CO_2. Infectious viral titers in culture supernatants were assessed at 1 h, 24 h, 48 h, 72 h, and 96 h post-infection (hpi) by titration in Vero E6 cells. One set of

bronchus and lung tissues were fixed at 96 hpi in 10% formalin and processed for immunohistochemistry staining.

2-Dimensional Differentiated Human Airway Organoid Culture and Infection

The human airway organoids were established from human lung tissues, as previously described (*5*). The 2-dimensional differentiated airway organoid model was further built on the airway organoids, as previously described (*6*), with a few modifications. In brief, airway organoids were dissociated into single cells using TrypLE select (GIBCO, https://www.thermofisher.com) for 10 min at 37°C. The digest was then sheared by using 25-gauge needle and strained over a 40 μm cell strainer. We seeded 150,000 cells onto Transwell insert (Corning, https://www.corning.com) pre-coated with rat tail collagen 1 (Corning). The cells were cultured in a mixture of airway organoid growth medium (*5*) and Pneumacult-ALI (Stemcell, https://www.stemcell.com) complete base medium at a ratio of 1:1 at 37°C for 3–4 days. Once the cells reached confluency, they were cultured at air-liquid interface (ALI) in Pneumacult-ALI Maintenance Medium (Stemcell). The medium was changed every 3 days. The 2-dimensional transwell cultures were used for infection after 3 weeks of differentiation. Cells were infected with the coronaviruses at a multiplicity of infection of 2 at the apical side for 1 h at 37°C. Cells were washed with PBS and culture at ALI in the same growth medium.

Viral Titration by $TCID_{50}$ assay

Vero-E6 cells were seeded on 96-well tissue culture plates 1 day before the virus titration ($TCID_{50}$) assay. Cells were washed once with PBS and replenished with 2% DMEM medium supplemented with 100 units/mL of penicillin and 100 μg/mL of streptomycin. Serial dilutions of virus supernatant, from 0.5–7 log, were performed before adding the virus dilutions onto the plates in quadruplicate. The plates were observed daily for cytopathic effects. The endpoint of viral dilution leading to CPE in 50% of inoculated wells was estimated by using the Karber method (*7*). Area-under-curve (AUC) was calculated by integrating infectious virus titers at 24–96 hpi in ex vivo bronchus and lung tissues.

Immunohistochemical Staining

Immunohistochemical staining of the respiratory tract tissue was carried out for the SARS-CoV-2 nucleoprotein, as previously described (*2*). The fixed paraffin-embedded ex vivo cultures of human tissues were stained with SARS-CoV nucleoprotein (4D11) (*4*,*5*,*8*). The tissue

sections were first microwaved in 10 mmol citrate buffer, blocked with 10% normal horse serum at room temperature. The sections were then incubated with 4D11 antibody followed by alkaline phosphatase (AP) conjugated antimouse antibody (Vector Laboratories, Inc., https://vectorlabs.com) and developed with VectorRed (VR) (Vector Laboratories, Inc.).

Real-time PCR assay

The RNA of infected cells were extracted at 48 h post infection by using a MiniBEST Universal RNA Extraction Kit (TaKaRa Bio, Inc., http://www.takara-bio.com). RNA was reverse-transcribed by using Oligo-dT primers with Advantage RT-for-PCR Kit (TaKaRa Bio, Inc.). mRNA expression of target genes was performed by using ViiA7 Real-Time PCR System (Applied Biosystems, https://www.thermofisher.com). The gene expression profiles were quantified and normalized with β-actin, as previously described (*9–11*).

Cytometric Bead Array

Protein concentration of interferon gamma-induced protein-10 in the supernatants collected from apical and basolateral chambers was determined by bead-based immunoassays, BD Cytometric Bead Array (BD Bioscience, https://www.bdbiosciences.com) according to the manufacturer's protocol. In brief, 50 µL of cell culture supernatant and a 10-point standard curve (ranging from 0–2,500 pg/mL) was used for the measurement of each cytokine and chemokine. The samples were analyzed by using a BD LSR Fortessa Analyzer (BD Bioscience). Standard curves for the cytokines and chemokines were built and the fluorescence intensity concentrations were calculated by using FlowJo version 7.6.1 (https://www.flowjo.com).

Biosafety and Ethics

All infection work was carried out in a Biosafety Level-3 facility. Informed consent was obtained from all subjects and approval was granted by the Institutional Review Board of the University of Hong Kong and the Hospital Authority (Hong Kong West) (approval no. UW 20–167).

References

1. Sit THC, Brackman CJ, Ip SM, Tam KWS, Law PYT, To EMW, et al. Infection of dogs with SARS-CoV-2. Nature. 2020;586:776–8. PubMed https://doi.org/10.1038/s41586-020-2334-5

2. Hui KPY, Cheung MC, Perera RAPM, Ng KC, Bui CHT, Ho JCW, et al. Tropism, replication competence, and innate immune responses of the coronavirus SARS-CoV-2 in human respiratory tract and conjunctiva: an analysis in ex-vivo and in-vitro cultures. Lancet Respir Med. 2020;8:687–95. PubMed https://doi.org/10.1016/S2213-2600(20)30193-4

3. Chan MC, Chan RW, Yu WC, Ho CC, Yuen KM, Fong JH, et al. Tropism and innate host responses of the 2009 pandemic H1N1 influenza virus in ex vivo and in vitro cultures of human conjunctiva and respiratory tract. Am J Pathol. 2010;176:1828–40. PubMed https://doi.org/10.2353/ajpath.2010.091087

4. Hui KP, Chan LL, Kuok DI, Mok CK, Yang ZF, Li RF, et al. Tropism and innate host responses of influenza A/H5N6 virus: an analysis of ex vivo and in vitro cultures of the human respiratory tract. Eur Respir J. 2017;49:1601710. PubMed https://doi.org/10.1183/13993003.01710-2016

5. Hui KPY, Ching RHH, Chan SKH, Nicholls JM, Sachs N, Clevers H, et al. Tropism, replication competence, and innate immune responses of influenza virus: an analysis of human airway organoids and ex-vivo bronchus cultures. Lancet Respir Med. 2018;6:846–54. PubMed https://doi.org/10.1016/S2213-2600(18)30236-4

6. Lamers MM, Beumer J, van der Vaart J, Knoops K, Puschhof J, Breugem TI, et al. SARS-CoV-2 productively infects human gut enterocytes. Science. 2020;369;50–4. PubMed https://doi.org/10.1126/science.abc1669

7. Karber G. 50% end-point calculation. Arch Exp Pathol Pharmak. 1931;162:480–3.

8. Nicholls JM, Butany J, Poon LL, Chan KH, Beh SL, Poutanen S, et al. Time course and cellular localization of SARS-CoV nucleoprotein and RNA in lungs from fatal cases of SARS. PLoS Med. 2006;3:e27. PubMed https://doi.org/10.1371/journal.pmed.0030027

9. Chan RW, Chan MC, Wong AC, Karamanska R, Dell A, Haslam SM, et al. DAS181 inhibits H5N1 influenza virus infection of human lung tissues. Antimicrob Agents Chemother. 2009;53:3935–41. PubMed https://doi.org/10.1128/AAC.00389-09

10. Hui KP, Lee SM, Cheung CY, Mao H, Lai AK, Chan RW, et al. H5N1 influenza virus-induced mediators upregulate RIG-I in uninfected cells by paracrine effects contributing to amplified cytokine cascades. J Infect Dis. 2011;204:1866–78. PubMed https://doi.org/10.1093/infdis/jir665

11. Hui KP, Lee SM, Cheung CY, Ng IH, Poon LL, Guan Y, et al. Induction of proinflammatory cytokines in primary human macrophages by influenza A virus (H5N1) is selectively regulated by IFN regulatory factor 3 and p38 MAPK. J Immunol. 2009;182:1088–98. PubMed https://doi.org/10.4049/jimmunol.182.2.1088

Appendix Table 1. Cases causing the fourth wave of coronavirus disease, Hong Kong

Cluster	Patient	Date RT-PCR–confirmed
Hotel A	A1*	20 Sep 2020
Hotel A	A2*	20 Sep 2020
Hotel A	A3*	20 Sep 2020
Hotel A	A4*	4 Oct 2020
Hotel B	B1*	20 Sep 2020
Hotel B	B2*	20 Sep 2020
Bar/Build X	BB1*	1 Oct 2020
Bar/Build X	BB2*	3 Oct 2020
Bar/Build X	BB3*	4 Oct 2020
Bar/Build X	BB4	4 Oct 2020
Bar/Build X	BB5*	5 Oct 2020
Bar/Build X	BB6*	6 Oct 2020
Bar/Build X	BB7*	7 Oct 2020
Bar/Build X	BB8*	7 Oct 2020
Bar/Build X	BB9*	7 Oct 2020
Bar/Build X	BB10*	8 Oct 2020
Bar/Build X	BB11*	9 Oct 2020
Bar/Build X	BB12*	10 Oct 2020
Bar/Build X	BB13*	11 Oct 2020
Bar/Build X	BB14	12 Oct 2020
Bar/Build X	BB15	13 Oct 2020
Hotel C	C1*	6 Oct 2020
Hotel C	C2*	9 Oct 2020
Hotel C	C3*	9 Oct 2020
Hotel C	C4*	9 Oct 2020
Hotel C	C5*	9 Oct 2020
Hotel C	C6*	10 Oct 2020
Hotel C	C7*	12 Oct 2020
Hotel C	C8*	18 Oct 2020
Hotel C	C9*	19 Oct 2020

*Sequenced samples

Appendix Table 2. Names, accession numbers, and submission dates of severe acute respiratory syndrome coronavirus 2 genomes deposited in GISAID and used in a study of outbreaks in the fourth wave of coronavirus disease, Hong Kong

Virus name	Accession No.	Date collected 2020	Originating laboratory	Submitting laboratory	Authors
Australia/VIC1158/2020	EPI_ISL_430602	9 Apr	Victorian Infectious Diseases Reference Laboratory (VIDRL)	Microbiological Diagnostic Unit Public Health Laboratory and Victorian Infectious Diseases Reference Laboratory, The Peter Doherty Institute for Infection and Immunity	L. Caly et al.
Australia/VIC1787/2020	EPI_ISL_456649	27 May	Victorian Infectious Diseases Reference Laboratory (VIDRL)	Microbiological Diagnostic Unit Public Health Laboratory and Victorian Infectious Diseases Reference Laboratory, Doherty Institute	Caly et al.
Australia/VIC98/2020	EPI_ISL_419810	16 Mar	Victorian Infectious Diseases Reference Laboratory (VIDRL)	Victorian Infectious Diseases Reference Laboratory and Microbiological Diagnostic Unit Public Health Laboratory, Doherty Institute	Caly et al.
England/20132080404/2020	EPI_ISL_423805	24 Mar	Respiratory Virus Unit, Microbiology Services Colindale, Public Health England	Respiratory Virus Unit, Microbiology Services Colindale, Public Health England	M. Galiano et al.
England/CAMB-77909/2020	EPI_ISL_439431	31 Mar	Department of Pathology, University of Cambridge	Wellcome Sanger Institute for the COVID-19 Genomics UK (COG-UK) consortium	L.W. Meredith et al.
England/NORW-EC85D/2020	EPI_ISL_457566	13 May	Quadram Institute Bioscience	COVID-19 Genomics UK (COG-UK) Consortium	D.J. Baker et al.
England/SHEF-C03E5/2020	EPI_ISL_420221	29 Mar	Virology Department, Sheffield Teaching Hospitals NHS Foundation Trust	Department of Infection, Immunity and Cardiovascular Disease, The Florey Institute, The Medical School, University of Sheffield	Thushan de Silva et al.
France/HDF-3668/2020	EPI_ISL_443316	25 Mar	CH CompiÃgne Laboratoire de Biologie	National Reference Center for Viruses of Respiratory Infections, Institut Pasteur, Paris	M. Albert et al.
HongKong/VB20175856/2020	EPI_ISL_539823	14 Aug	Communicable Disease Branch	Hong Kong Department of Health	Alan K.L. Tsang et al
HongKong/VM20066524/2020	EPI_ISL_539814	21 Jul	Tuen Mun Hospital	Hong Kong Department of Health	A.K.L. Tsang et al.
Iceland/99/2020	EPI_ISL_417768	10 Mar	The National University Hospital of Iceland	deCODE genetics	D.F. Gudbjartsson et al.
India/GJ-GBRC137b/2020	EPI_ISL_458095	24 May	B.J. Medical College and Civil hospital	Gujarat Biotechnology Research Centre	R. Kumar et al.
India/OR-RMRC164/2020	EPI_ISL_455764	7 May	REGIONAL VRDL, ICMR-RMRC BBSR	Immunogenomics lab, Institute of Life Sciences, Bhubaneswar	S. Raghav et al.
Japan/Hu_DP_Kng_19–031/2020	EPI_ISL_420889	14 Feb	Takayuki Hishiki Kanagawa Prefectural Institute of Public Health	Takayuki Hishiki Kanagawa Prefectural Institute of Public Health	Hishiki et al.
Nepal/2097/2020	EPI_ISL_754068	30 Jul	Nepal Korea Friendship Municipality Hospital	Nepal Health Research Council	P. Gyanwali et al.
Nepal/2178/2020	EPI_ISL_754069	31 Jul	Nepal Korea Friendship Municipality Hospital	Nepal Health Research Council	P. Gyanwali et al.
Nepal/2673/2020	EPI_ISL_754070	7 Aug	Nepal Korea Friendship Municipality Hospital	Nepal Health Research Council	P. Gyanwali et al.
Nepal/459A/2020	EPI_ISL_754072	3 Aug	National Public Health Laboratory	Nepal Health Research Council	P. Gyanwali et al.
Nepal/61/2020	EPI_ISL_410301	13 Jan	National Influenza Centre, National Public Health Laboratory, Kathmandu, Nepal	The University of Hong Kong	R. Sah et al.

Virus name	Accession No.	Date collected 2020	Originating laboratory	Submitting laboratory	Authors
Nepal/614/2020	EPI_ISL_754062	7 Aug	Kathmandu University	Nepal Health Research Council	P. Gyanwali et al.
Nepal/616/2020	EPI_ISL_754063	10 Aug	Kathmandu University	Nepal Health Research Council	P. Gyanwali et al.
Nepal/617/2020	EPI_ISL_754064	10 Aug	Kathmandu University	Nepal Health Research Council	P. Gyanwali et al.
Nepal/618/2020	EPI_ISL_754065	10 Aug	Kathmandu University	Nepal Health Research Council	P. Gyanwali et al.
Nepal/619/2020	EPI_ISL_754066	10 Aug	Kathmandu University	Nepal Health Research Council	P. Gyanwali et al.
Nepal/620/2020	EPI_ISL_754067	9 Aug	Kathmandu University	Nepal Health Research Council	P. Gyanwali et al.
Nepal/70N/2020	EPI_ISL_754073	3 Aug	National Public Health Laboratory	Nepal Health Research Council	P. Gyanwali et al.
Nepal/962/2020	EPI_ISL_754060	3 Aug	National Public Health Laboratory	Nepal Health Research Council	P. Gyanwali et al.
Nepal/C3031/2020	EPI_ISL_754071	10 Aug	Nepal Korea Friendship Municipality Hospital	Nepal Health Research Council	P. Gyanwali et al.
Nepal/H353/2020	EPI_ISL_754061	3 Aug	National Public Health Laboratory	Nepal Health Research Council	P. Gyanwali et al.
NewZealand/20VR3045/2020	EPI_ISL_456402	25 Apr	Wellington SCL	Institute of Environmental Science and Research (ESR)	M. Storey et al.
Portugal/PT0163/2020	EPI_ISL_453879	28 Mar	Unknown	Instituto Nacional de Saude (INSA)	Borges et al.
Spain/MD-ISCIII-201738/2020	EPI_ISL_419237	7 Mar	Fundacion Jimenez Diaz	Instituto de Salud Carlos III	Iglesias-Caballero et al.
USA/CA-CZB-1237/2020	EPI_ISL_454657	12 May	County of Santa Clara Public Health Department	Chan-Zuckerberg Biohub	CZB Cliahub Consortium et al.
Wales/PHWC-3180E/2020	EPI_ISL_446492	11 Apr	Wales Specialist Virology Centre	Public Health Wales Microbiology Cardiff	C. Moore et al.
Wales/PHWC-32B6E/2020	EPI_ISL_446656	14 Apr	Wales Specialist Virology Centre	Public Health Wales Microbiology Cardiff	C. Moore et al.
Wuhan/HB-WH1–131/2020	EPI_ISL_454910	2 Mar	Wuhan Chain Medical Labs (CMLabs)	State Key Laboratory of Biotherapy of Sichuan University	B. Du et al.
Wuhan/WIV04/2019	EPI_ISL_402124	30 Dec 2019	Wuhan Jinyintan Hospital	Wuhan Institute of Virology, Chinese Academy of Sciences	P. Zhou et al.

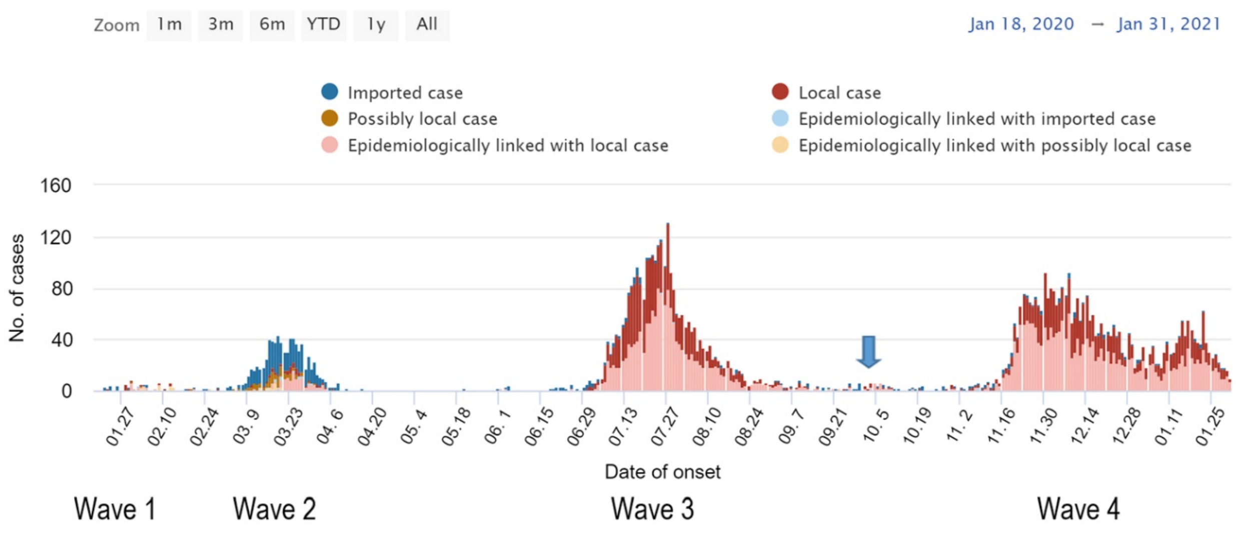

Appendix Figure 1. Coronavirus disease epidemic waves, Hong Kong, China. Hong Kong experienced 4 waves of COVID-19. The number of cases per day are show with differential epidemiologic links are indicated. Blue arrow indicates outbreaks in bar/building X and hotel C. Data were extracted from https://covid19.sph.hku.hk.

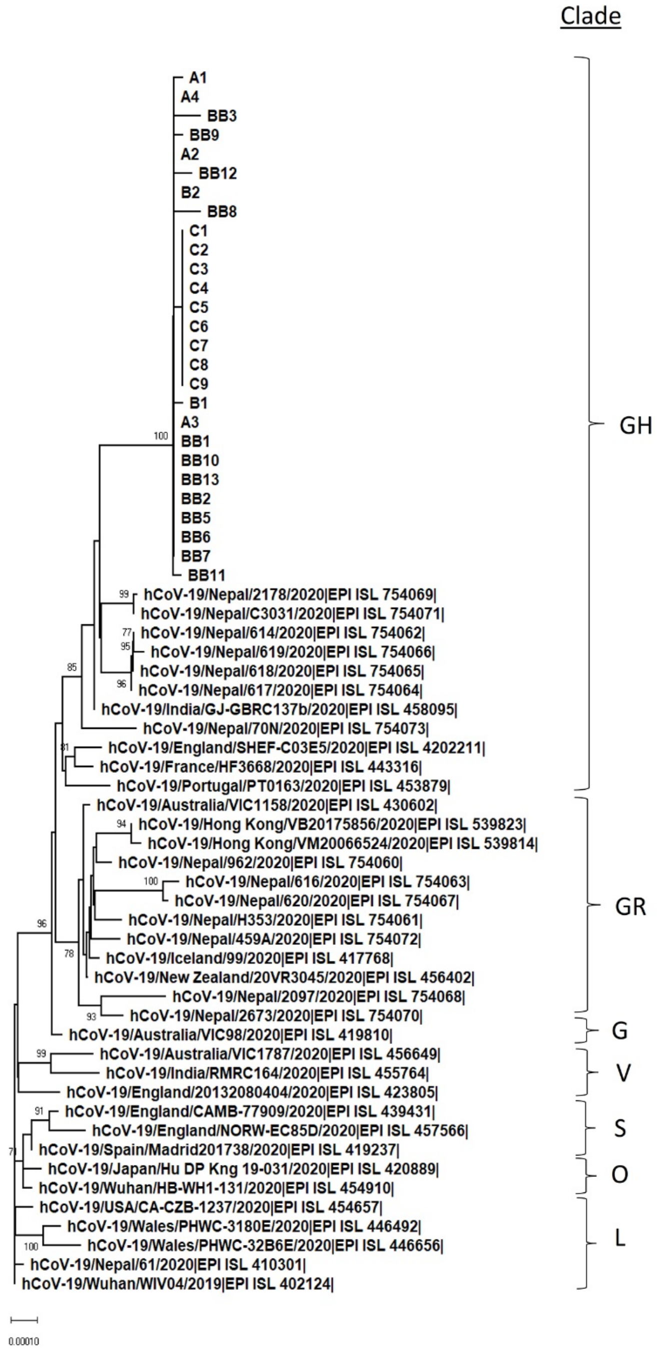

Appendix Figure 2. Phylogenetic tree of the SARS-CoV-2 detected in COVID-19 cases causing the fourth epidemic wave, Hong Kong, China. We used human SARS-CoV-2 WIV04 as the root of this phylogenetic tree. The tree was constructed by using the neighbor-joining method. Only bootstrap values >70 are shown. Viruses from clades L, S, V, G, GH, GR, and O (others) are included in the analysis. EPI ISL accession nos. for sequences were retrieved from GISAID (https://platform.gisaid.org). Scale bar indicates estimated genetic distance. COVID-19, coronavirus disease. SARS-CoV-2, severe acute respiratory syndrome coronavirus 2.

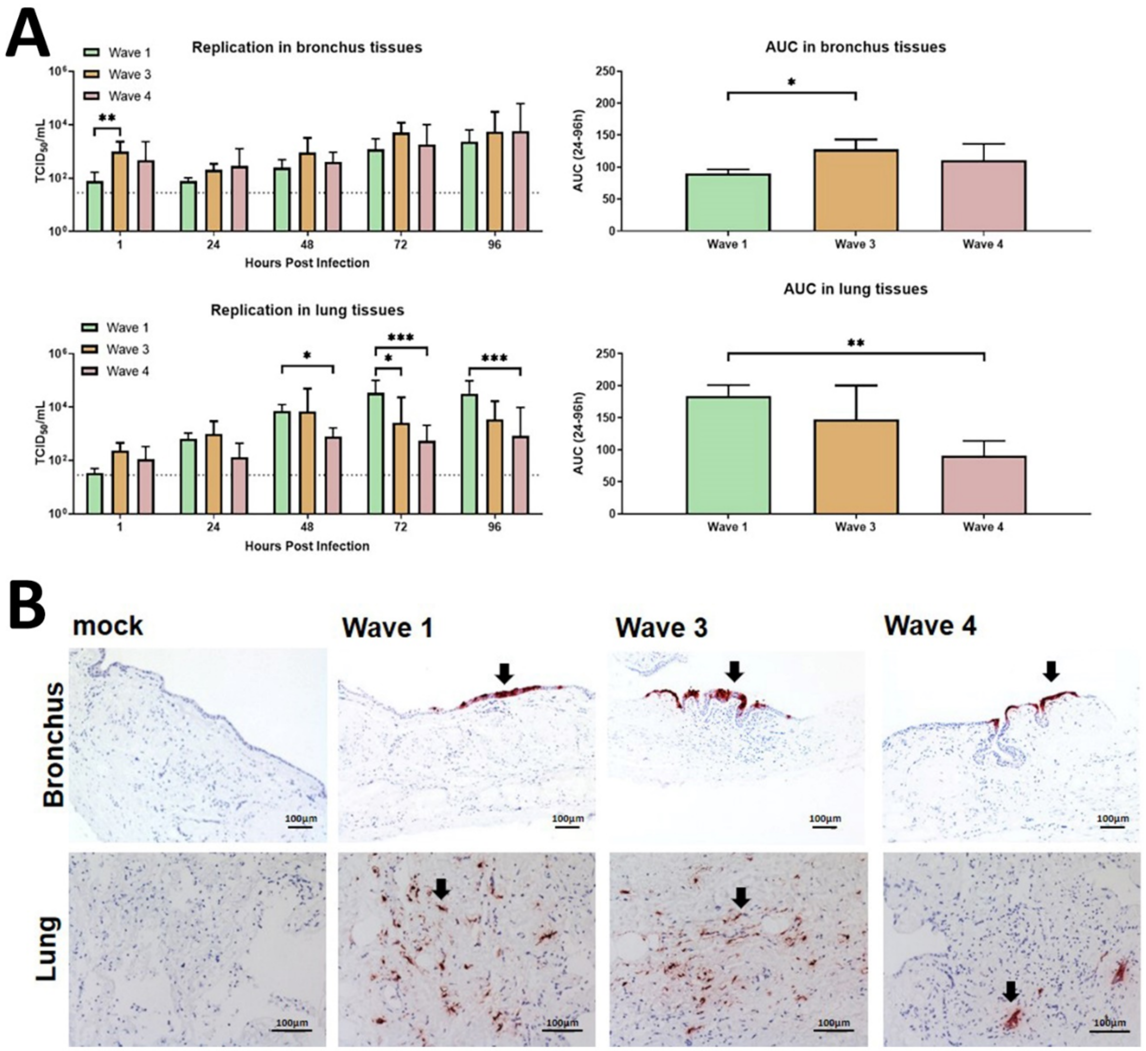

Appendix Figure 3. Viral replication kinetics of wave 1, 3, and 4 SARS-CoV-2 viruses in ex vivo cultures of human respiratory tract tissues. Human ex vivo cultures of bronchus (n = 4) and lung (n = 3), were infected with 5×10^5 $TCID_{50}$/mL at 37°C. Culture supernatants were harvested at the indicated times and virus titers were measured by $TCID_{50}$ assay. A) Bar-charts show the mean virus titer $\pm$ SD. The horizontal dotted line denotes the limit of detection in the $TCID_{50}$ assay. Area-under-curve (AUC) was calculated from the viral titers from 24h to 96h. Bar charts of AUC show the mean $\pm$ SD. The differences between viral titers were compared using 2-way ANOVA followed by a Tukey multiple-comparison test. The differences of AUC between viruses were compared using 1-way ANOVA followed by a Tukey multiple-comparison test. *p<0.05; **p<0.01; ***p<0.001. B) Mock and infected tissues formalin-fixed at 96 h post infection. Top row, bronchi; bottom row, lung. Paraffin embedded sections were subjected to immunohistochemical staining with a monoclonal antibody against the SARS-CoV-2 NP. Arrows indicate positive cells are in red. Magnification ×100 in bronchus tissues; magnification ×200 in lung tissues. Scale bar indicates 100 µm. NP, nucleoprotein; SARS-CoV-2, severe acute respiratory syndrome coronavirus 2; SD, standard deviation.

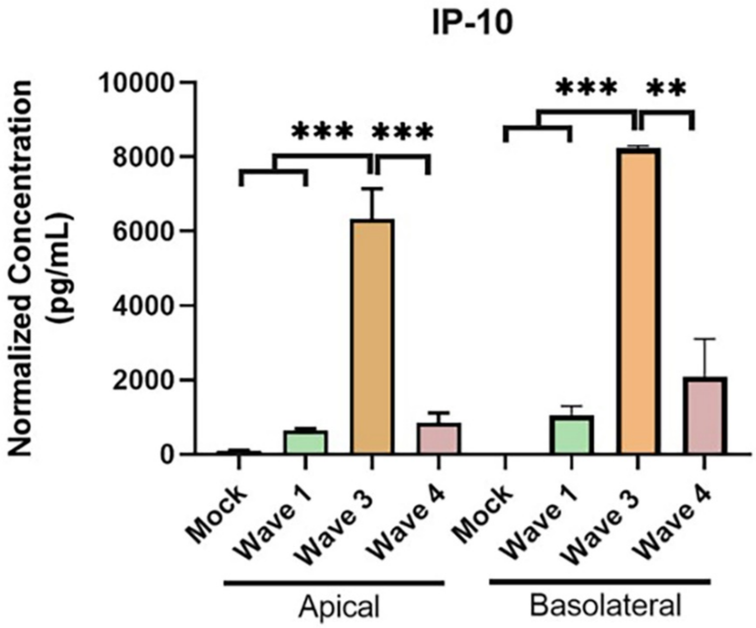

Appendix Figure 4. IP-10 expression in human airway organoids infected by wave 1, 3, or 4 SARS-CoV-2 virus, Hong Kong, China. At 48 h post infection, concentrations of IP-10 in the culture supernatants from both apical and basal chamber were measured by cytometric bead assay. The concentrations of infected cells were normalized with ORF1b gene. Results are the calculated mean from 2 independent experiments ± SD of mean. The differences were compared using 1-way ANOVA followed by a Tukey multiple-comparison test. Means and standard deviation error bars are as shown **p<0.01; ***p<0.001. IP-10, interferon gamma-induced protein-10 SARS-CoV-2, severe acute respiratory syndrome coronavirus 2.
